# The Smartphone App haMSter for Tracking Patient-Reported Outcomes in People With Multiple Sclerosis: Protocol for a Pilot Study

**DOI:** 10.2196/25011

**Published:** 2021-05-07

**Authors:** Patrick Altmann, Werner Hinterberger, Fritz Leutmezer, Markus Ponleitner, Tobias Monschein, Tobias Zrzavy, Gudrun Zulehner, Barbara Kornek, Rupert Lanzenberger, Klaus Berek, Paulus Stefan Rommer, Thomas Berger, Gabriel Bsteh

**Affiliations:** 1 Department of Neurology Medical University of Vienna Vienna Austria; 2 CAKE Communications Vienna Austria; 3 Department of Psychiatry and Psychotherapy Medical University of Vienna Vienna Austria; 4 Department of Neurology Medical University of Innsbruck Innsbruck Austria

**Keywords:** mHealth, mobile health, remote monitoring, patient-reported outcomes, multiple sclerosis, telemedicine

## Abstract

**Background:**

Treatment and monitoring decisions in people with multiple sclerosis (MS) are based commonly on clinician-reported outcomes. These reflect physical and radiological disease activity and are the most relevant endpoints in clinical trials. Over the past few years, the number of studies evaluating so-called patient-reported outcomes (PROs) has been increasing. PROs are reports from patients concerning their own health perception. They are typically obtained by means of questionnaires and aim to quantify symptoms such as fatigue, depression, and sexual dysfunction. The emergence of PROs has made a tremendous contribution to understanding the individual impact of disease in people with MS and their health-related quality of life. However, the assessment of PROs consumes resources, including time and personnel. Thus, useful ways to conveniently introduce PROs into clinical practice are needed.

**Objective:**

We aim to provide a rationale and pilot study protocol for a mobile health (mHealth) solution named “haMSter” that allows for remote monitoring of PROs in people with MS.

**Methods:**

The core function of haMSter is to provide three scientifically validated PRO questionnaires relevant to MS for patients to fill out at home once a month. Thereby, longitudinal and remote documentation of PROs is enabled. A scoring algorithm graphically plots PRO scores over time and makes them available at the next visit.

**Results:**

The pilot study is currently ongoing and will evaluate adherence to this mHealth solution in 50 patients over a period of 6 months. Results from the haMSter pilot study are expected in 2021.

**Conclusions:**

haMSter is a novel mHealth-based solution for modern PRO research, which may constitute the first step in achieving the ability to integrate PROs in clinical practice. This allows for a more problem-oriented approach in monitoring visits, which addresses patient needs and ultimately saves time.

**Trial Registration:**

ClinicalTrials.gov NCT04555863; https://clinicaltrials.gov/ct2/show/NCT04555863

**International Registered Report Identifier (IRRID):**

DERR1-10.2196/25011

## Introduction

### Multiple Sclerosis and Patient-Reported Outcomes

Multiple sclerosis (MS) is a chronic neurological disease associated with inflammation and neurodegeneration. Its worldwide prevalence is estimated at 2.5 million people affected, and it is the most common cause of disability in young adults, aside from trauma [1-3]. Disease monitoring in people with MS encompasses continuous evaluation of the following three major components: clinical disease activity, radiological disease activity, and, albeit mostly for research purposes, biomarkers [4,5]. These outcomes can be summarized as clinician-reported outcomes. They represent the mainstay in determining the disease course and treatment response in routine MS care and are also the main endpoints in clinical trials. Complementary to clinician-reported outcomes, so-called patient-reported outcomes (PROs) are reports from patients concerning their own health perception, quantifying either specific symptoms that are hard to objectify, such as fatigue, depression, and sexual dysfunction, or more general parameters, such as quality of life, working abilities, treatment adherence, and treatment satisfaction. Over the past few years, PROs have contributed to displaying the disease burden in MS and its impact on health-related quality of life. So far, several PRO measures (PROMs) for MS have been validated and established, such as scores for quality of life, fatigue, and sexual functioning [6,7].

While garnering information about a patient’s subjective impairment to health through PROs would theoretically have a palpable benefit, application in routine care is greatly hindered. Currently, filling out and evaluating paper-based PRO questionnaires are consuming a considerable amount of resources in terms of time and personnel. Thus, useful ways to conveniently introduce PROs into clinical practice are needed.

### Mobile Health and Multiple Sclerosis

The use of mobile health (mHealth) may offer strategies for easily administered PRO questionnaires. This could facilitate research and advance the focus from merely reporting disease parameters to understanding an individual’s impairment caused by disease. So far, a recognizable effort has been made to offer mHealth technologies for managing MS. These solutions can be summarized as elements of (1) screening and assessment, (2) treatment and rehabilitation, (3) advice and education, and (4) disease monitoring and management [8]. However, only a handful of these tools focus on integrating validated PROs into clinical practice. One smartphone app, for example, offers remote and active testing of *surrogate* markers for dexterity and mobility that correlated well with on-site administered tests [9]. Another study investigated an on-site tablet-based method for evaluating bladder control [10]. The most prominent mHealth solution for remote patient monitoring in people with MS is probably the Multiple Sclerosis Performance Test. It is a tablet-based tool designed to measure physical disability, and it has spurred a lot of interest and subsequent adaptions [11,12]. Nevertheless, solutions that allow remote tracking of well-established PROMs in MS have not yet been reported.

### Study Aim

The aim of this report is to introduce *haMSter* as an mHealth-based solution for tracking PROs in people with MS remotely. Through the use of the haMSter app, patients are offered an opportunity to fill out PRO questionnaires anywhere and anytime they feel comfortable. The scoring algorithm of haMSter provides neurologists a unique opportunity to visualize the longitudinal course of their patients’ PROs. A pilot study evaluating the feasibility of the haMSter protocol is currently ongoing.

## Methods

### Study Design

This study protocol will be used in an uncontrolled pilot trial evaluating adherence to the haMSter app in 50 people with MS. Upon informed consent to participate in this study, eligible patients will be able to download the app on their smartphones during a regular visit to our MS clinic and use it at home over a period of 6 months.

### Study Setting

**Figure 1:**
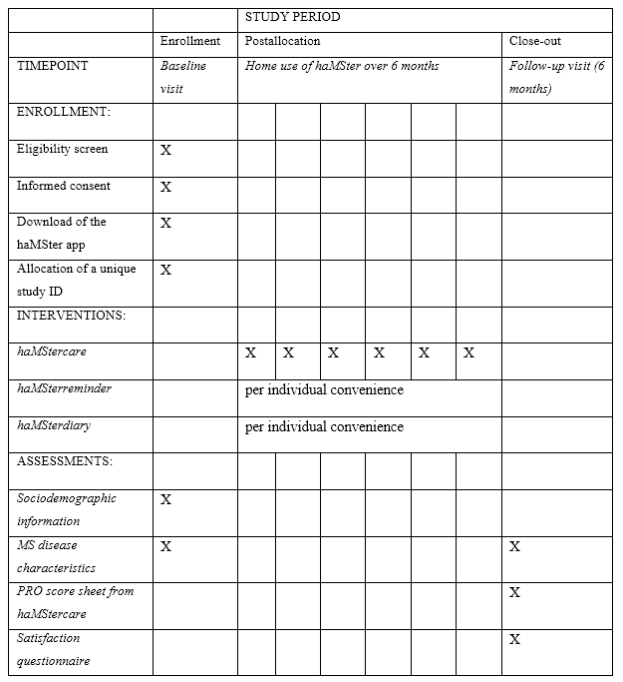
Overview of study enrollment, interventions, and assessments over the course of the haMSter pilot trial (Standard Protocol Items: Recommendations for Interventional Trials [SPIRIT] checklist). MS: multiple sclerosis; PRO: patient-reported outcome.

This is a monocentric study at the MS Clinic of the Department of Neurology, Medical University of Vienna, Austria. An outline of the study protocol is shown in Figure 1 [13]. Patients are instructed to fill out three specific PRO questionnaires (“haMStercare”) on their smartphone once every 30 days. In addition, patients are introduced to two other features of haMSter, which are available at their own convenience. These are a reminder for taking their medication (“haMSterreminder”) and a diary (“haMSterdiary”). At the follow-up visit, a Bluetooth printer in the exam room will allow the patient to print out a PRO score sheet that graphically shows the score for each PRO over the course of the study. This illustration is intended as a base for discussing symptoms and the patient’s individual health perception. The study concludes with the administration of a paper-based satisfaction questionnaire including a validated outcome measure for satisfaction with telemedicine (Telemedicine Perception Questionnaire [TMPQ] [14]) and an empirical questionnaire. The latter collects user feedback regarding technical implementation, design, handling, and possibilities for further improvement of the app’s utility.

### haMSter Smartphone App

#### haMStercare: PRO Questionnaires

**Figure 2:**
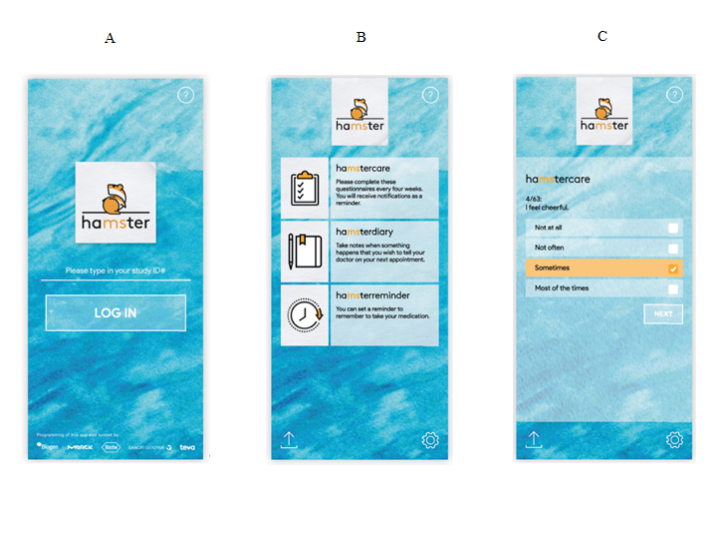
Screenshots of the the login screen (A), main menu screen (B), and a sample patient-reported outcome (PRO) question (C). The screenshots provided here have been translated from German to English for the purpose of this article. The lower end of the log-in screen reads “Programming of this app was funded by” and lists the corporate logos of pharmaceutical companies that provided funding for programming the haMSter app.

The core function of haMSter is the provision of three validated and well-established PROMs in MS. After obtaining written consent from the respective PRO proprietor, we included three different questionnaires that are commonly used in MS research and for which a German version is available. The first is the Hospital Anxiety and Depression Scale (HADS), which is a widely used screening tool for depression and anxiety [15]. It reports two separate scores for depression and anxiety. The second is the Multiple Sclerosis Impact Scale (MSIS-29), which is a survey of quality of life measures in people with MS that provides subscores for physical and psychological impairment in context with MS [16]. The third is the Fatigue Scale for Motor and Cognitive Functions (FSMC), which is an MS-specific scale that quantifies motor and cognitive fatigue. The haMStercare feature is programmed to provide a pop-up message to remind patients every 30 days to fill out the questionnaires. Figure 2 illustrates the login screen, the main menu that navigates through the three haMSter features, and an example question from the HADS.

#### haMSterreminder: Reminder for Medication

The haMSter app allows participants to set a reminder for their medication. All medications approved for the treatment of MS in Austria as of April 1, 2019 (when the app was programmed) can be selected by the user. These include alemtuzumab, cladribine, dimethyl fumarate, fingolimod, glatiramer acetate, interferon-beta preparations, natalizumab, ocrelizumab, and teriflunomide. The reminder’s time algorithm is based on the fixed dosing regimen for these medications. Patients are made aware that this reminder does not intend to replace their own responsibility for adhering to their medication.

#### haMSterdiary: Diary Entries for Patients

**Figure 3:**
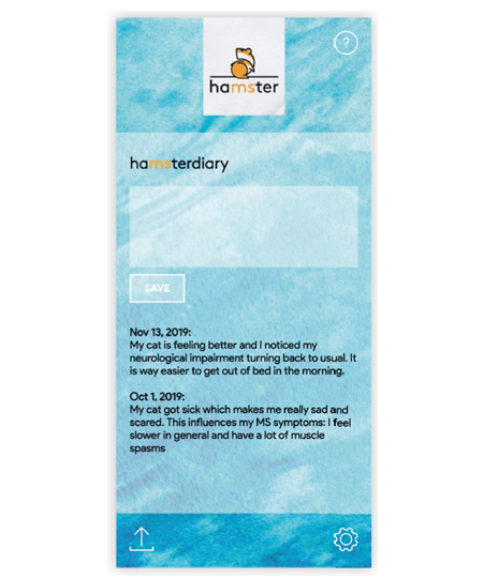
Sample entry for haMSterdiary. The screenshot provided here has been translated from German to English for the purpose of this article.

Another feature of the haMSter app is a diary notepad (Figure 3). Patients can type in any matter concerning their lives with MS, such as certain events or symptoms they wish to communicate to their doctors at the next visit. The haMSter diary saves these entries together with the current date.

#### Coding Process

The haMSter app was coded using the WebView-based Cordova app (Apache Software Foundation). The Cordova native API (application programming interface) served as the app’s main framework. User input was programmed to not communicate with any server. Data entered into the haMSter app is stored as an HTML (hypertext markup language) web storage API using a JavaScript Object Notation (JSON; Oracle Corporation) based on the “JSON.parse” and “JSON.stringify” commands. Repeated testing for plausibility and reliability of the haMSter app’s performance was implemented before release and over a period of 3 months. This was performed using the PhoneGap command-line interface version 9.0.0 (PhoneGap CLI; Apache Software Foundation) through a node package manager, the developer tool in google chrome (Google International LLC), and prototype devices operating on Android version 8.0.0 or above (Google International LLC) and Apple iOS version 5.0.0 or above (Apple Inc).

#### Considerations of Data Protection

The pilot study using the haMSter protocol adheres to current data protection guidelines in Austria as of April 1, 2019. Patients are educated on these guidelines on the informed consent form, which has been approved by our local ethics review board. In short, the main considerations of data protection regarding the use of haMSter are (1) the app works exclusively offline, (2) no patient-specific data are entered into the app, and (3) data are stored nowhere else but on the patient’s smartphone. The app itself only operates on a study identification number that is assigned upon enrollment.

### Participants

#### Eligibility Criteria

The pilot study evaluating haMSter will enroll any patient fulfilling the current diagnostic criteria for MS [17], regardless of their age, disease phenotype, or medication. Inclusion is offered to patients owning a smartphone and expressing a will to participate. Exclusion criteria are obvious language barriers.

#### Recruitment Procedure

Patients will be informed about this study during any visit to our MS outpatient clinic. The study will be advertised as an investigation into the feasibility of a smartphone app determining patient-reported symptoms electronically and repeatedly over time. All patients expressing interest will receive a more detailed introduction to the app and its functions. Subsequently, the recruiting neurologist will accurately inform participants on relevant considerations regarding data protection (see above). Upon informed consent, patients can download the app on any device with an Apple or Android-based operating system. After successful download, the recruiting neurologist will enter relevant baseline characteristics (sociodemographic information and MS disease–specific characteristics) in a case report form on site.

### Funding Statements

The programming of the haMSter app has been funded through an unrestricted grant by pharmaceutical companies marketing medication for the treatment of MS. A study outline was sent out to all companies with branches in Austria at that time (Bayer, Biogen, Merck, Novartis, Roche, Sanofi-Genzyme, and Teva-Ratiopharm). Out of these seven companies, five agreed to cover the costs for programming the app (Biogen, Merck, Roche, Sanofi-Genzyme, and Teva-Ratiopharm Austria). A sponsorship agreement was signed between the inventor of the app and a sponsoring company’s representative. Funding parties were not involved in any decisions regarding any content of the app, the study protocol and design, or any matter concerning the pilot study itself at any point in time. As for the PRO questionnaires included in haMSter, the proprietors of the German versions of the HADS and FSMC received financial compensation for the use of their questionnaires in the pilot study and signed a licensing agreement. The amount of this compensation was calculated based on the frequency of access to the respective questionnaire (ie, 50 patients, once a month over 6 months). The author of the German version of the MSIS-29 kindly offered its use for free.

### Timeline

The idea for the haMSter app was conceived in November 2018. The coding process began in May 2019 and was finished in October 2019. After a period of beta testing and some delay due to the COVID-19 pandemic, the pilot study was launched in April 2020 (“first patient in”), and it is expected to end in April 2021 (“last patient out”).

## Results

### Baseline and Sociodemographic Characteristics

To provide an overview of the patients participating in this pilot study, we will report typical baseline and sociodemographic characteristics documented at baseline and follow-up. The structure is provided in Textbox 1 [18].

**Sociodemographic and clinical characteristics of patients to be determined in this pilot study.**

**Parameter**

- Participants analyzed, n (%)
  Age (years), mean (SD)
- Sex, n (%)
  - Female
  - Male

- Disease phenotype, n (%)
  - Relapsing multiple sclerosis (MS)
  - Progressive MS

- Expanded Disability Status Scale
  - Median
  - 0-3.5
  - 4 or above

- Number of relapses
  - Last 12 months

- Disease duration (years), n (%)
- MS medication, n (%)
  - Moderately effective
  - Highly effective
  - No treatment
  - Other medication and comorbidities

- Family status, n (%)
  - Single
  - Relationship
  - Married
  - Number of children

- Education, n (%)
  - Nine or less years of schooling
  - Secondary schooling
  - College degree

### Primary Outcome Measure

**Figure 4:**
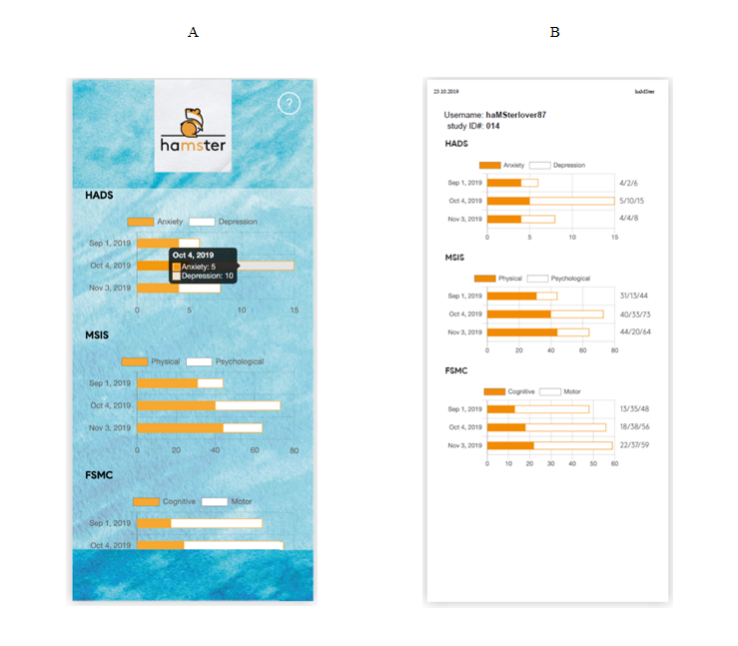
Screenshots of the patient-reported outcome (PRO) scoring information on the smartphone (A) and a printed score sheet (B). The screenshots provided here have been translated from German to English for the purpose of this article.

The primary outcome measure is the adherence to filling out the questionnaires once per month. Results will be displayed as the mean percentage of filled out questionnaires based on the PRO scoring sheet. Figure 4 illustrates a sample scoring sheet as viewed in the app and as a printout that is presented to the patient and discussed with the physician during the end of the study visit. We will investigate whether adherence decreases over time by using the Friedman test and Jonckheere-Terpstra test for monotonic trend.

### Exploratory Outcome Measures

Exploratory outcome measures include patient perceptions of this new mHealth method based on mean TMPQ values. There will also be a further exploratory analysis of PROs in conjunction with MS disease characteristics and of the empirical questionnaires with respect to the utility and design of haMSter. If reasonable, we will perform a subgroup analysis to identify patients with high (75th percentile) and low (25th percentile) satisfaction. Another secondary goal is group comparisons of adherence between patients with and those without upper extremity disability as defined by the Expanded Disability Status Scale (EDSS) score.

### Statistical Considerations

Currently, there is no consensus on a significant threshold for adherence to mHealth methods. With respect to our main outcome measure, we regard a minimum adherence of 50% or higher as relevant (participants completed the three PRO questionnaires [a total of 63 questions] three times over a period of 6 months). As for the exploratory outcome measures, we aim to provide descriptive statistics (categorical variables expressed as frequencies and percentages, and continuous variables tested for normal distribution by the Kolmogorow-Smirnow test and presented as mean and standard deviation or median and interquartile range as appropriate).

### Ethics Review and Trial Registration

The ethics committee at the Medical University of Vienna, Austria, approved this study in September 2019 (EK1798/2019). Written informed consent will be obtained from each patient, and the study protocol follows the guidelines set by the Declaration of Helsinki. The trial has been registered at ClinicalTrials.gov (identifier: NCT04555863).

## Discussion

This is the protocol for a pilot study testing a new smartphone app named haMSter. This mHealth-based approach enables continuous measurement of PROs in people with MS and uses scientifically validated questionnaires. Textbox 2 outlines the hypotheses regarding the possible future implications of haMSter and its use in patient care. We believe the haMSter app can save time during routine consultations and improve quality of face-to-face visits with respect to targeting *patient-specific problems* rather than discussing a variety of *possible problems*. On a separate note, repeated questionnaires, such as the ones included in haMSter, may reflect the patients’ everyday reality compared with a single assessment. Even so, since this is a pilot trial, the implementation of this app in real life may call for further adjustments.

**Future roles of haMSter in patient-centered care.**

- haMSter makes monitoring of patient-reported outcomes (PROs) convenient and easy.

Advantage: Researchers do not have to resort to paper-based questionnaires anymore.

Future implication: PRO research can advance to a longitudinal dimension.

- haMSter demonstrates advances in modern PRO research.

Advantage: PRO research becomes accessible more easily.

Future implication: This may influence treatment considerations in the future.

- haMSter gives room to underrepresented symptoms.

Advantage: The haMSter protocol may unveil symptoms that were previously unnoticed.

Future implication: Health-related quality of life can improve further.

- haMSter is a solution to save resources in clinical care.

Advantage: Through a graphical illustration, PROs can easily be interpreted in context with time.

Future implication: Focus in routine care is diverted to a more patient-oriented discussion.

Results from the haMSter pilot study are expected in 2021. The primary endpoint is adherence to this new method based on the frequency of completed questionnaires over the study period. Furthermore, the utility of haMSter will be carefully assessed through the use of satisfaction questionnaires. The selection process for this study may introduce bias, as only patients with an a priori acceptance of this method would participate. However, we will report on the number of patients declining participation and their given reasons for it.

Taken together, this pilot study is set to lay the foundation for further advancement of haMSter as a digital solution with the ultimate goal of setting a new standard for modern and personalized patient care. Future possibilities of evolving this app further will be explored.

## Abbreviations

EDSS: Expanded Disability Status Scale
FSMC: Fatigue Scale for Motoric and Cognitive Fatigue
HADS: Hospital Anxiety and Depression Scale
MS: multiple sclerosis
MSIS-29: Multiple Sclerosis Impact Scale
PRO: patient-reported outcome
PROM: patient-reported outcome measure
TMPQ: Telemedicine Perception Questionnaire
